# Modeling glucose and free fatty acid kinetics in glucose and meal tolerance test

**DOI:** 10.1186/s12976-016-0036-3

**Published:** 2016-03-02

**Authors:** Yanjun Li, Carson C. Chow, Amber B. Courville, Anne E. Sumner, Vipul Periwal

**Affiliations:** Laboratory of Biological Modeling, National Institute of Diabetes and Digestive and Kidney Diseases (NIDDK), National Institutes of Health (NIH), MSC 5621, LBM, NIDDK, NIH, Bethesda, MD 20892-5621 USA; Nutrition Department, Clinical Center, National Institute of Diabetes and Digestive and Kidney Diseases (NIDDK), National Institutes of Health (NIH), Bethesda, MD 20892 USA; Diabetes, Endocrinology, and Obesity Branch, National Institute of Diabetes and Digestive and Kidney Diseases (NIDDK), National Institutes of Health (NIH), Bethesda, MD 20892 USA

**Keywords:** Glucose tolerance test, Lipolysis, Appearance rate

## Abstract

**Background:**

Quantitative evaluation of insulin regulation on plasma glucose and free fatty acid (FFA) in response to external glucose challenge is clinically important to assess the development of insulin resistance (World J Diabetes 1:36–47, 2010). Mathematical minimal models (MMs) based on insulin modified frequently-sampled intravenous glucose tolerance tests (IM-FSIGT) are widely applied to ascertain an insulin sensitivity index (IEEE Rev Biomed Eng 2:54–96, 2009). Furthermore, it is important to investigate insulin regulation on glucose and FFA in postprandial state as a normal physiological condition. A simple way to calculate the appearance rate (Ra) of glucose and FFA would be especially helpful to evaluate glucose and FFA kinetics for clinical applications.

**Methods:**

A new MM is developed to simulate the insulin modulation of plasma glucose and FFA, combining IM-FSIGT with a mixed meal tolerance test (MT). A novel simple functional form for the appearance rate (Ra) of glucose or FFA in the MT is developed. Model results are compared with two other models for data obtained from 28 non-diabetic women (13 African American, 15 white).

**Results:**

The new functional form for Ra of glucose is an acceptable empirical approximation to the experimental Ra for a subset of individuals. When both glucose and FFA are included in FSIGT and MT, the new model is preferred using the Bayes Information Criterion (BIC).

**Conclusions:**

Model simulations show that the new MM allows consistent application to both IM-FSIGT and MT data, balancing model complexity and data fitting. While the appearance of glucose in the circulation has an important effect on FFA kinetics in MT, the rate of appearance of FFA can be neglected for the time-period modeled.

**Electronic supplementary material:**

The online version of this article (doi:10.1186/s12976-016-0036-3) contains supplementary material, which is available to authorized users.

## Background

Insulin is one of the primary factors regulating plasma glucose and free fatty acid (FFA). The development of insulin resistance and type 2 diabetes (T2D) is closely associated with the abnormal regulation of insulin on carbohydrate metabolism. Dysfunction of whole body lipid metabolism plays an important role in the development of T2D [1–4]. The modulation of glucose and FFA metabolism by insulin depends on race, obesity, sex, and regional body fat distribution [5–7]. Quantitation of insulin regulation of glucose and FFA, including kinetic interactions in response to external physiological stimuli, is clinically important because of the potential to inform treatment paradigms for T2D.

Mathematical indexes have been defined to assess the effects of insulin on carbohydrate metabolism [8]. The insulin sensitivity index (*S*_*I*_) is the most frequently used, as directly measured by glucose clamp [9], or indirectly by a mathematical MM analysis of data from the insulin-modified frequently-sampled intravenous glucose tolerance test (IM-FSIGT) [10–13]. In addition, the FSIGT MM assesses the dynamic relationship between insulin and glucose, and is used clinically.

This approach has well-known limitations [8]. First and foremost, most MMs consider only insulin and glucose [14, 15]. Impaired lipid metabolism is related to the development of insulin resistance [16], so the regulation of lipid metabolism by insulin must be assessed. While some MMs have investigated lipid dynamics [17–19], parameters in these models are often based on mechanisms with scant physiological support. Neither glucose clamp nor FSIGT is a “normal” state, which limits physiological insights from these approaches. Therefore, we aim to quantify whole body regulation by insulin of carbohydrate and lipid metabolism simultaneously in response to meals. Secondly, the insulin-glucose system is obviously connected so using measured insulin as an external input in the model is conceptually suspect. The insulin-modified FSIGT does have an administered bolus of exogenous insulin to assess insulin sensitivity independent of endogenous insulin secretion through the MM parameters. Nevertheless, an ideal model would be autonomic, and starting from an initial measurement of insulin and glucose, would predict the subsequent time-course of both variables, given subject-specific parameters for such a model. However, such an autonomic model would also apply only to a state with no meals ingested. Clearly, this would not address the former limitation of the MM approach.

In the postprandial state, dietary glucose and FFA enters the circulation after digestion and absorption [20, 21]. Their rates of appearance (Ra) have to be taken into account. Although Ra can be measured by isotope tracer technique [22], this is expensive, not always feasible, and typically provides sparse data. Mathematical approaches have been applied to evaluate glucose and FFA kinetics in the postprandial state. Some studies modeled glucose responses in oral glucose tolerance test (OGTT) or meal test (MT) [23–27]. The Ra of glucose was introduced with a complex algorithm and many parameters, limiting clinical utility. Some models for postprandial FFA metabolism [28, 29], however, did not consider Ra of FFA.

Indeed, the FSIGT and the mixed meal models are trying to fit complex physiological processes with simplified models involving just a few parameters. For example, the FSIGT ignores hepatic glucose output as an independent process. The aim, of course, is to ascribe some overall biological relevance to these few parameters, as has been done for decades with the insulin sensitivity index. Our aim in this paper is to figure out if a simple Ra function can actually model the complex physiology of food ingestion and absorption through the gastro-intestinal tract, when used along with the intravenous glucose model. The point is that if the Ra combined with the intravenous glucose model is inadequate for the MT, this inadequacy will show up as an additional contribution to the Ra of glucose, and hence as a bad fit with the functional parameterization of the Ra. A comparison of the adequacy of this Ra functional parameterization for two ethnic groups is a test of the general applicability of this simplified physiology in this controlled setting.

The specific aims of our study are: first, to develop a new MM to evaluate insulin regulation of glucose and FFA in both IM-FSIGT and MT together; and second, to develop a simplified empirical function to parametrize the Ra of glucose or FFA in MT so that contributions of Ra on glucose or FFA responses can be quantitatively evaluated with no change in the equations from the FSIGT equations. This may be helpful to evaluate Ra of glucose or FFA in a postprandial state clinically as a simpler, albeit limited, alternative to tracer studies.

The model augments a previously developed model of the response of FFA and glucose to insulin in IM-FSIGT alone [30]. Experimental data from two ethnic groups – African American women (AA) and white women are studied. The contributions of Ra of glucose and FFA on corresponding glucose or FFA kinetics in MT are evaluated with different definitions of Ra in various simulation combinations, and compared with another two published models. The parameter values for each group obtained in each model are determined and compared.

## Methods

### Subjects

Premenopausal women - 13 African-Americans (AA) and 15 white matched for age and body mass index (BMI) participated (Additional file 1: Table S1). Recruitment was by flyers, newspaper advertisements, and the National Institutes of Health web site. Only nondiabetic women with normal hemograms, liver, kidney, and thyroid function were enrolled. Participants were not taking medications known to affect either glucose or lipid metabolism, or oral contraceptives. Because the menstrual cycle does not impact FFA metabolism, studies were performed throughout the cycle [7, 31]. The Institutional Review Board of the National Institute of Diabetes and Digestive and Kidney Diseases approved the study. All subjects gave informed consent.

### Experimental protocol and measurement

#### IM-FSIGT

The experimental design of IM-FSIGT is as in previous work [5, 7, 30]. Each subject underwent an insulin modified frequently sampled intravenous glucose tolerance test (IM-FSIGT). The IM-FSIGT was performed in the morning after a 12-h overnight fast. Intravenous catheters were placed in both antecubital veins. Arterialized blood samples were collected for analysis. At time 0, dextrose (0.3 g/kg) was injected over 1 min and a bolus injection of insulin (0.03 U/kg) was given at 20 min. Samples for glucose, insulin, and FFA were obtained at −10, −1, 0, 1, 2, 3, 4, 5, 6, 7, 8, 10, 12, 14, 16, 20, 22, 23, 24, 25, 27, 30, 40, 50, 60, 70, 80, 90, 100, 120, 150, and 180 min.

#### MT

The experimental design of meal test (MT) has been described in detail previously [32]. After a 7-day diet equilibration period [32], in which all of subjects’ meals were provided through a metabolic kitchen, subjects were provided with standardized breakfast. The standardized breakfast consisted of 30 % of the participants’ energy needs on the last day of the equilibration period, 20 % protein, 40 % fat and 40 % carbohydrate. Plasma samples for glucose, insulin and FFA were obtained at 0, 30, 60, 90, 120, 150, 180, 210, 240, 270, 300, 330 and 360 min. Triglyceride (TG) were also measured at 0, 120, 240, 360 min.

Glucose and TG were measured on Dimension Vista 1500 analyzers (Siemens) using standard automated methods. Insulin was measured using an immunochemiluminometric assay from Diagnostic Products on an Immulite 2500 machine (Diagnostic Products). FFA was measured with Wako HR Series NEFA-HR kit (Wako Diagnostics, Wako Chemicals USA, Inc., Richmond, VA) and run on a COBAS FARA-II analyzer (Roche Diagnostics, Indianapolis, IN). Physiological variables associated with anthropometrics (e.g., waist, hip, and thigh circumferences), VAT and SAT, body composition etc. were measured as in previous studies [7].

Insulin resistance represented by insulin sensitivity index (S_I_) was determined from the MM, given below. The acute insulin responses to glucose (AIRg) in IM-FSIGT was calculated as the incremental area under the curve (AUC) for insulin between 0 and 10 min for the insulin concentration above basal level. The disposition index (DI), calculated as the product of AIRg of IM-FSIGT and S_I_, was determined as a measure of β-cell function, specifically defined as the ability of the circulating insulin concentration to compensate for insulin resistance. Statistical analysis was performed with Statistica (www.statsoft.com). The null hypothesis is there is no difference between AA women and white women. The critical value is α = 0.05.

### Model description

#### Model structure

The new model (MOD 1, detailed in Additional file 1, Section B) is partly based on our previous study [30]. Briefly, the main modification is that 1) in the dynamic equation of plasma FFA, it is assumed that insulin can stimulate the FFA clearance [33–37] as well; 2) rate of appearance (Ra) terms are introduced in the dynamic equations for glucose or FFA in meal test (MT), representing the net appearance of glucose or FFA into circulation via digestive system after a meal.

To thoroughly investigate the performance of the present model and the influence of Ra on glucose or FFA dynamics in MT, this model was compared with two published models –MOD 2 [30] and MOD 3 [29]. In brief, MOD 1 and MOD 2 have the same dynamic mass balance equation for glucose and insulin kinetics. They are distinguished in FFA clearance. FFA kinetics equation has two terms - lipolysis and clearance. MOD 2 assumes the constant clearance rate. MOD 1 assumes clearance is insulin-dependent. Both MOD 1 and MOD 2 assume that insulin modulates glucose and FFA via the same insulin action (*X*, or remote insulin compartment). MOD 3 directly uses insulin with specific time delay for lipolysis and clearance, respectively. The appearance rate (Ra) of glucose or FFA in MT is introduced under specific simulation combinations. The components of the three models are compared in Table 1. The descriptions of all parameters are listed in Additional file 1: Table S2.

**Table 1:** Comparison of each component of three models

	MOD 1	MOD 2	MOD 3
Glucose kinetics	*S*_*G*_ *G*_*b*_ − (*S*_*G*_ + *S*_1_ *X*)*G*		-
Insulin kinetics	*C*_*x*_[*I*(*t*) − *X* − *I*_*bx*_]		-
FFA lipolysis	${l}_0+\frac{l_2}{1+{\left(X/{X}_2\right)}^{A_{lipo}}}$	${l}_0+\left[\frac{l_2}{1+{\left(X/{X}_2\right)}^{A_l}}\right]$	$\frac{V_m^{Lip}}{1+{\left(I\left(t-{t}_{DelayLip}\right)/{K}_{Lip}\right)}^{h_{lip}}}$
FFA clearance	${C}_f\left[1+\frac{{\left(X/{K}_{Cl}\right)}^{A_{Cl}}}{1+{\left(X/{K}_{Cl}\right)}^{A_{Cl}}}\right]F$	*C*_*f*0_ *F*	${k}_{\mathrm{Re}m}F+\frac{V_m^{\mathrm{Re}m}{I}^{h_{rem}}\left(t-{t}_{Delay\mathrm{R}\mathrm{e}m}\right)}{K_m^{h_{\mathrm{Re}m}}+{\left(I\left(t-{t}_{Delay\mathrm{R}\mathrm{e}m}\right)/{K}_{\mathrm{Re}m}\right)}^{h_{\mathrm{Re}m}}}F$

*G* is plasma glucose, *F* is plasma FFA, *I* is plasma insulin as model input, *X* is the insulin action representing the indirect effect of insulin on the regulation of plasma glucose and FFA. The detailed descriptions of parameters are listed in Additional file 1: Table S2. Note that *I*_*bx*_ is a fitted parameter that is determined through the action of *X* on glucose and FFA. Thus, it is not necessarily the same as measured basal insulin, and we have used the subscript *bx* to emphasize this fact

#### Appearance rate of glucose and FFA

To avoid too many parameters and complex algorithms, empirical functional forms were used to simplify the expression and simulation of Ra. According to experimental measurement [38, 39] and other mathematical studies [23, 27], two different equations for Ra were used.

Type I is similar to a log-normal function:

1 $$ R{a}_G(t)=\left\{\begin{array}{cc}\hfill 0\hfill & \hfill \mathrm{t}=0\hfill \\ {}\hfill \frac{\Delta_G}{t{\sigma}_G} \exp \left\{-\left[\frac{{\left[ \ln \left(t/{m}_G\right)\right]}^2}{2{\sigma}_G^2}\right]\right\}\kern1em \hfill & \hfill \mathrm{otherwise}\hfill \end{array}\right. $$

2 $$ R{a}_F(t)=\left\{\begin{array}{cc}\hfill 0\hfill & \hfill \mathrm{t}\le {t}_{0F}\hfill \\ {}\hfill \frac{\Delta_F}{\left(t-{t}_{0F}\right){\sigma}_F} \exp \left(-\left[\frac{{\left[ \ln \left(\left(t-{t}_{0F}\right)/{m}_F\right)\right]}^2}{2{\sigma}_F^2}\right]\right)\hfill & \hfill \mathrm{otherwise}\hfill \end{array}\right. $$

where *Δ*_*G*_, *Δ*_*F*_ are phenomenological magnitude factors, m_G_, m_F_ are time scale parameters, *σ*_*F*_ and *σ*_*G*_ are width parameters, and t_0F_ is the specific time at which FFA originating from chylomicron triglyceride appears in the plasma. The areas under the curves (AUC) of glucose and FFA Ra for different periods of MT were calculated. Two variables are fixed t_0F_ = 60; m_F_ = 300 based on a preliminary study.

Type II is from Pearson et al.’s study [40],

3 $$ R{a}_G(t)\kern0.5em =\frac{\phi_Gt}{\tau_G^2} \exp \left\{-{t}^2/\left(2{\tau}_G^2\right)\right\} $$

in which *ϕ*_*G*_ is the magnitude coefficient, and *τ*_*G*_ is the timescale coefficient. Both of them have to be determined, as compared to three parameters that need to be determined for the Type I glucose Ra.

#### Simulation combinations

The behaviors of the three models were compared under four simulation Combinations: 1) without considering Ra for glucose and FFA; 2) considering Ra of glucose and FFA expressed as Type I functions; 3) only Ra of glucose is incorporated expressed as a Type I function; 4) only Ra of glucose is incorporated, expressed as a Type II function [40]. While it seems obvious that excluding Ra for glucose and FFA (Combination 1 above) is unnecessary, we present it for completeness so that the difference between Combination 2 and Combination 3 can be clearly exhibited in the progression: no Ra, both Ra’s, only glucose Ra. To completely investigate the parameter stability, based on these results, additional combinations are involved for MOD 1 and MOD 2, S1) only IM-FSIGT; S2) only MT (Type I function); S3) only MT(Type II function). With experiment data from IM-FSIGT and MT in each subject, the individual parameters are determined for each model and each Combination, respectively. The simulations of glucose and FFA dynamics of each model and each Combination can then be compared. Moreover, Bayes information criterion (BIC) was applied to evaluate the simulation results of three models with each combination.

#### Calculation of Ra

Herrero et al.’s study [25] provided an alternative way to evaluate Ra needing somewhat more involved calculations. According to the glucose dynamic equation, Ra is function of glucose derivative and glucose concentration at specific time.

4 $$ R{a}_G(t)=\frac{d{G}_{MT}}{dt}-{S}_G{G}_b+\left({S}_G+{S}_I{X}_{MT}\right){G}_{MT} $$

in which *G*_*MT*_ is plasma glucose concentration in MT and *dG*_*MT*_*/dt* is the derivative; *X*_*MT*_ is the simulated insulin action in MT; *S*_*G*_, *S*_*I*_ and *G*_*b*_ are parameters. In the present study, because the insulin action (*X)* can be simulated via the models, Ra of glucose can be calculated directly. The parameter values are obtained in each combination condition, respectively. *dG*_*MT*_*/dt* is approximated based on the measured plasma glucose concentration at specific times in MT. Cubic splines were used to interpolate both glucose concentration and glucose derivatives with a time step of 1 min consistent with model simulations. Glucose Ra was calculated for each subject, for each Combination, respectively, and then the mean values of calculated Ra of glucose of each group were further compared with the mean values of model simulated Ra, respectively. The cubic spline used for the glucose concentration and glucose derivative introduces some artifacts, of course, but the results give a rough idea of the consistency of the simulated Ra functional forms with the observed experimental Ra.

### Parameter determinations

The unknown parameters corresponding to each models and each combination (detailed as above), were determined for each subject, respectively.

#### Objective function

The unknown parameters were determined for each subject, respectively, based on the following objective function.

5 $$ \underset{p}{\varSigma}\underset{m}{\varSigma}\underset{t}{\varSigma}\left[\frac{{\left({y}_{p,m,t}^{Data}-{y}_{p,m,t}^{Model}\right)}^2}{\sigma_{p,m}^2}\right] $$

where *p* ∈ (IM-FSIGT, MT) is the experimental protocol; *m* ∈ (G, F) is the measurement of glucose or FFA; *t* is the data at specific time in each case; σ^2^ is the variance, computed as described [30]. Briefly, to compute the variance, we used singular spectrum analysis with only one eigenvalue retained to find a continuous curve that approximated the data for each subject. The standard deviation of the data from this curve provides an estimate of the expected variance of the data from any model fit. The square of this standard deviation per time point is used as σ^2^. Thus this variance is a number that is estimated from the raw data, on a per subject basis. It is not optimized so it has no connection to the parameters that are being estimated using the minimization of the cost function.

Some parameters can be fixed according to experimental measurements. FFA from chylomicron triglyceride does not appear in the plasma until ~ 60 min. Therefore, in the equation of Ra of FFA, we fixed *t*_*0F*_ = 60 min. Moreover, model outputs were found to not be sensitive to some parameters according to our preliminary study, so we fixed *A*_*lipo*_ = 2; *A*_*Cl*_ *=* 2; *m*_*F*_ = 300 min. These simplifications did not influence goodness of fit.

#### Inputs and outputs

The inputs of the models are plasma insulin concentration as experimental measurements. The outputs are simulations of plasma glucose and FFA, which are compared with experimental data.

#### Initial state

The initial value of insulin action (*X*) is zero in both IM-FSIGT and MT. The initial condition of glucose or FFA in MT is the measured concentration at the initial time of the MT experiment. In IM-FSIGT, because only data within 10–180 min are considered, the initial value of glucose or FFA for simulation is the average concentration within 0–10 min for glucose or FFA, respectively.

#### Steady state

When *X* vanishes, steady state values are attained as all derivatives in the model equations are zero when insulin, glucose and FFA reach steady state values and Ra vanishes as well.

#### Model implementation

The models were implemented in Matlab Version 7.10 (R2010a) (The MathWorks Inc, Natick, MA, http://www.mathworks.com). Parameters were optimized by minimizing the objective function using FMINCON in Matlab. The constraints in optimization are introduced by setting the upper boundaries of parameters, which are chosen based on previous work [30] or a published model [29], typically 5 times the mean values. A change in the upper boundary had minor effects on parameter values. The tolerance is set to 10^−10^. The simulations are run on a computer (HP, Z400, Xeon®CPU, W3530, 2.8HGHz). In general, the simulation time for one individual is ~5 s when both IM-FSIGT and MT are considered.

## Results

The present study involves three models (MOD 1, new model; MOD 2 is from Periwal et al.’s study [30]; MOD 3 is from Ramos-Roman et al.’s study [29]). Simulations of each model were performed under four combinations, respectively, dependent on the introduction of glucose/FFA Ra or not and the expression for Ra used: 1) without considering Ra of glucose and FFA; 2) including both glucose and FFA Ra (Type I function); 3) only glucose Ra (Type I function); 4) only glucose Ra (Type II function [40]).

### Comparison of models for IM-FSIGT and meal test (MT)

The average simulated glucose or FFA of each group predicted by each model in IM-FSIGT and MT are compared with experimental data in Figs. 1, 2, 3 and 4, corresponding to simulation Combinations 1–4. The fractional residuals in each Combination are shown in Additional file 1: Figures S2–S5.

**Fig. 1:**
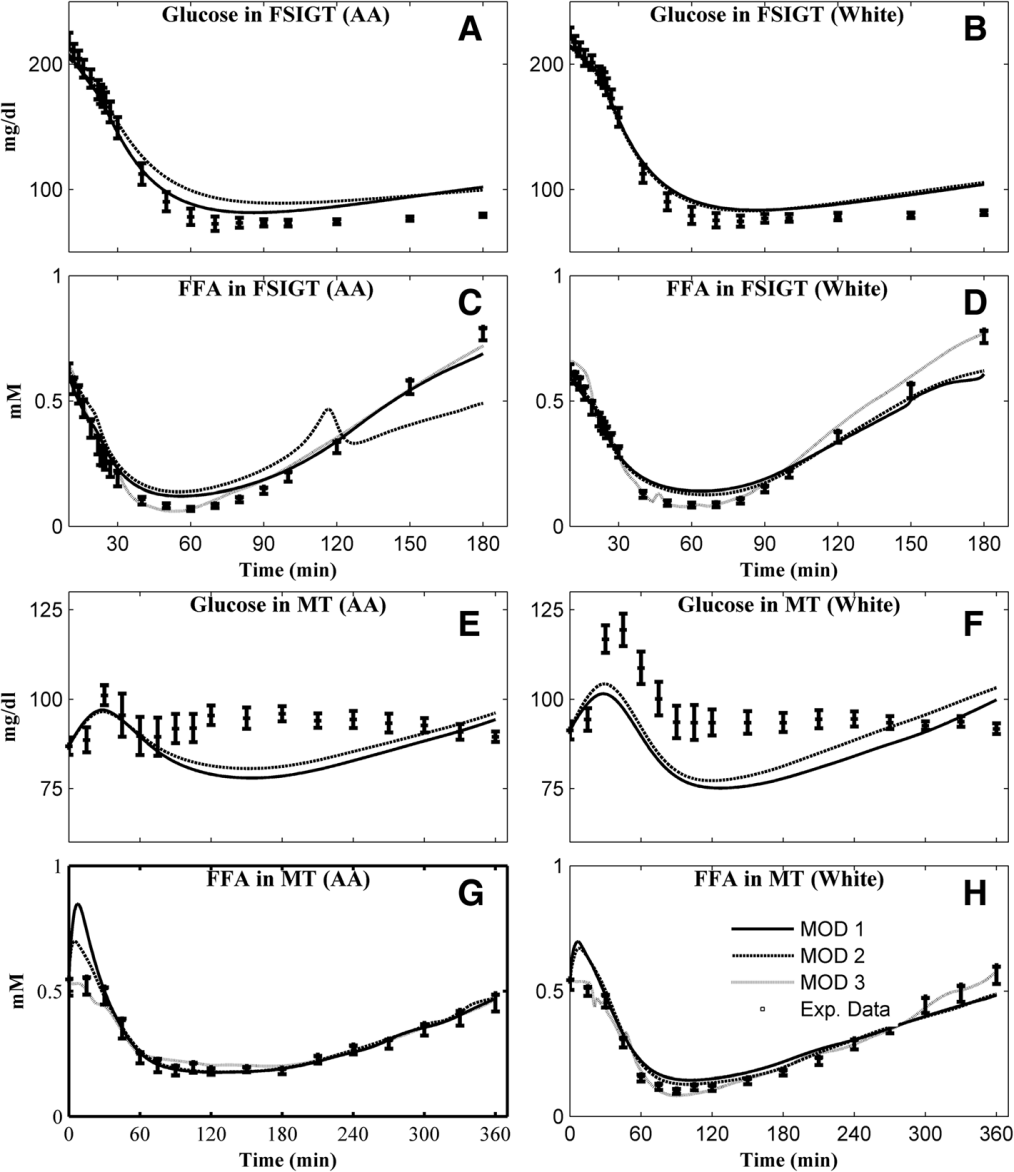
Mean individual simulations obtained in Combination 1 (without Ra of glucose and FFA) and comparison with the mean experimental data for Glucose in FSIGT of **a** AA women; **b** white women; FFA in FSIGT of **c** AA women; **d** white women; Glucose in MT of **e** AA women; **f** white women; FFA in MT of **g** AA women; **h** white women. *Open square* - experimental data (mean ± SE); *solid line* - mean simulation of MOD 1; *dashed line* - mean simulation of MOD 2; *dotted line* - mean simulation of MOD 3

**Fig. 2:**
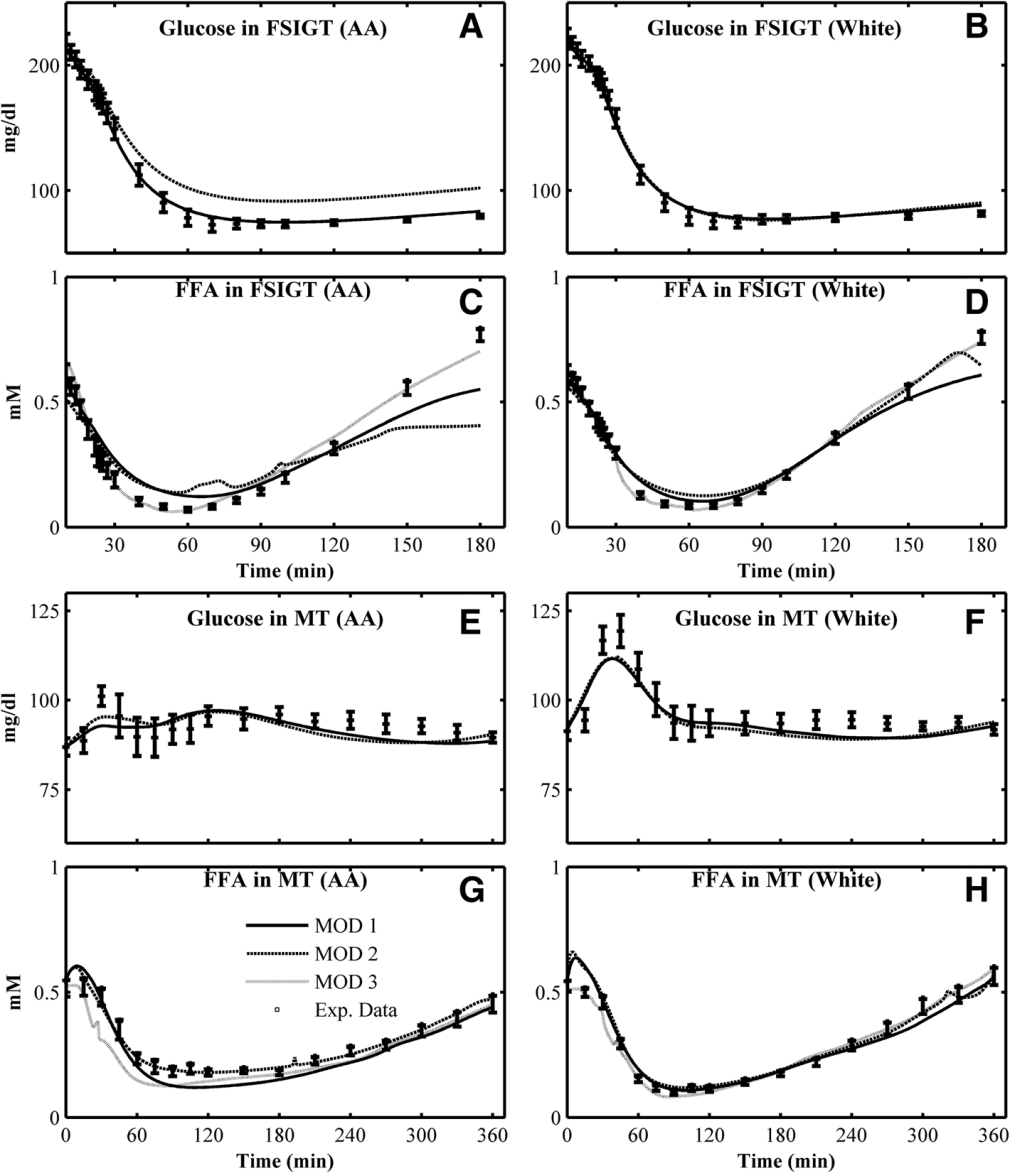
Mean individual simulations obtained in Combination 2 (considering Ra of glucose and FFA in MT) and comparison with the mean experimental data for Glucos in FSIGT of **a** AA women; **b** white women; FFA in FSIGT of **c** AA women; **d** white women; Glucose in MT of **e** AA; **f** white women; FFA in MT of **g** AA women; **h** white women. *Open square* - experimental data (mean ± SE); *solid line* - mean simulation of MOD 1; *dashed line* - mean simulation of MOD 2; *dotted line* - mean simulation of MOD 3

**Fig. 3:**
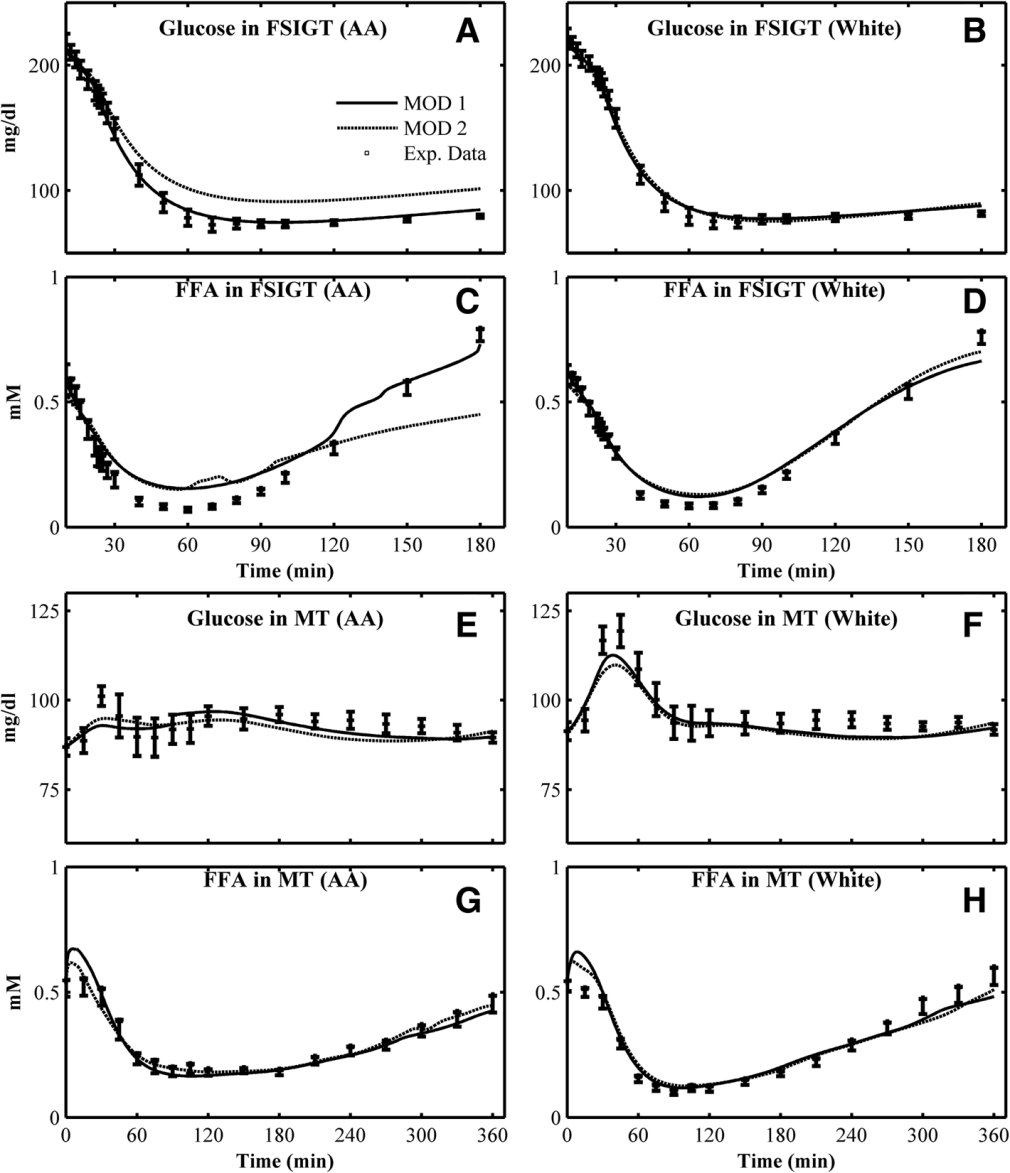
Mean individual simulations obtained in Combination 3 (with Ra of glucose only, Type I formula) and comparison with the mean experimental data for Glucose in FSIGT of **a** AA women; **b** white women; FFA in FSIGT of **c** AA women; **d** white women; Glucose in MT of **e** AA; **f** white women; FFA in MT of **g** AA women; **h** white women. *Open square* - experimental data (mean ± SE); *solid line* - mean simulation of MOD 1; *dashed line* - mean simulation of MOD 2

**Fig. 4:**
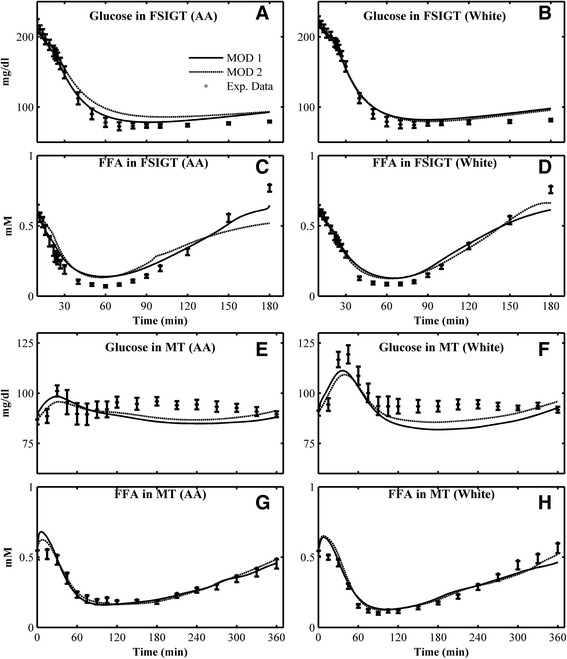
Mean individual simulations obtained in Combination 4 (with Ra of glucose only, Type II formula) and comparison with the mean experimental data for Glucose in FSIGT of **a** AA; **b** white women in FSIGT; FFA in FSIGT of **c** AA; **d** white women; Glucose in MT of **e** AA; **f** white women; FFA in MT of **g** AA women; **h** white women. *Open square* - experimental data (mean ± SE); *solid line* - mean simulation of MOD 1; *dashed line* - mean simulation of MOD 2

Without introducing Ra (Combination 1), the mean simulated glucose and FFA were compared with experimental data in Fig. 1, and the corresponding mean fraction residuals are shown in Additional file 1: Figure S2. In general, in IM-FSIGT, the simulated mean glucose or FFA responses are close to the data for all models (Fig. 1 a–d). However, although simulated FFA in MT still matches the data for the models (Fig. 1 g, h), the simulated glucose deviates from the data (Fig. 1 e, f).

After introducing Ra for both glucose and FFA (Combination 2), the mean predicted glucose and FFA responses of three models are compared with experimental data in Fig. 2. The predicted glucose in IM-FSIGT fit the data well (Fig. 2 a–b). The predicted glucose in MT is much closer to data than the simulation in Combination 1 (Fig. 2 e, f) and simulated time-course for white women (Fig. 2 f) matches experimental results better than for AA women in MT (Fig. 2 e). MOD 3 has better predictive power for FFA data in both tests, FSIGT and MT (Fig. 2 c–d, g–h).

Without considering FFA Ra, the effects of glucose Ra alone on glucose kinetics are compared in Figs. 3 and 4. Two kinds of glucose Ra functions are considered - Type I (Combination 3) or Type II (Combination 4). Using the Type I function (Combination 3), the mean prediction of glucose and FFA for MOD 1 and MOD 2 are compared with the mean experimental data of AA and white women in Fig. 3. In general, the model predictions of glucose for MOD 1 and MOD 2 are similar to the responses obtained in Combination 2 for both IM-FSIGT and MT. Without Ra of FFA, the fitting of FFA in MT even improved compared to the results from using both glucose and FFA rates of appearance, especially for MOD 2 (Fig. 3 e–f). If the Type II function for the glucose Ra is used (Combination 4), the mean model prediction of glucose in IM-FSIGT (Fig. 4 a–b,), and FFA in both of FSIGT and MT (Fig. 4 c–d, g–h) matches corresponding data. However, the simulated glucose responses in MT (Fig. 4 e–f) deviate from the data for both groups.

**Fig. 5:**
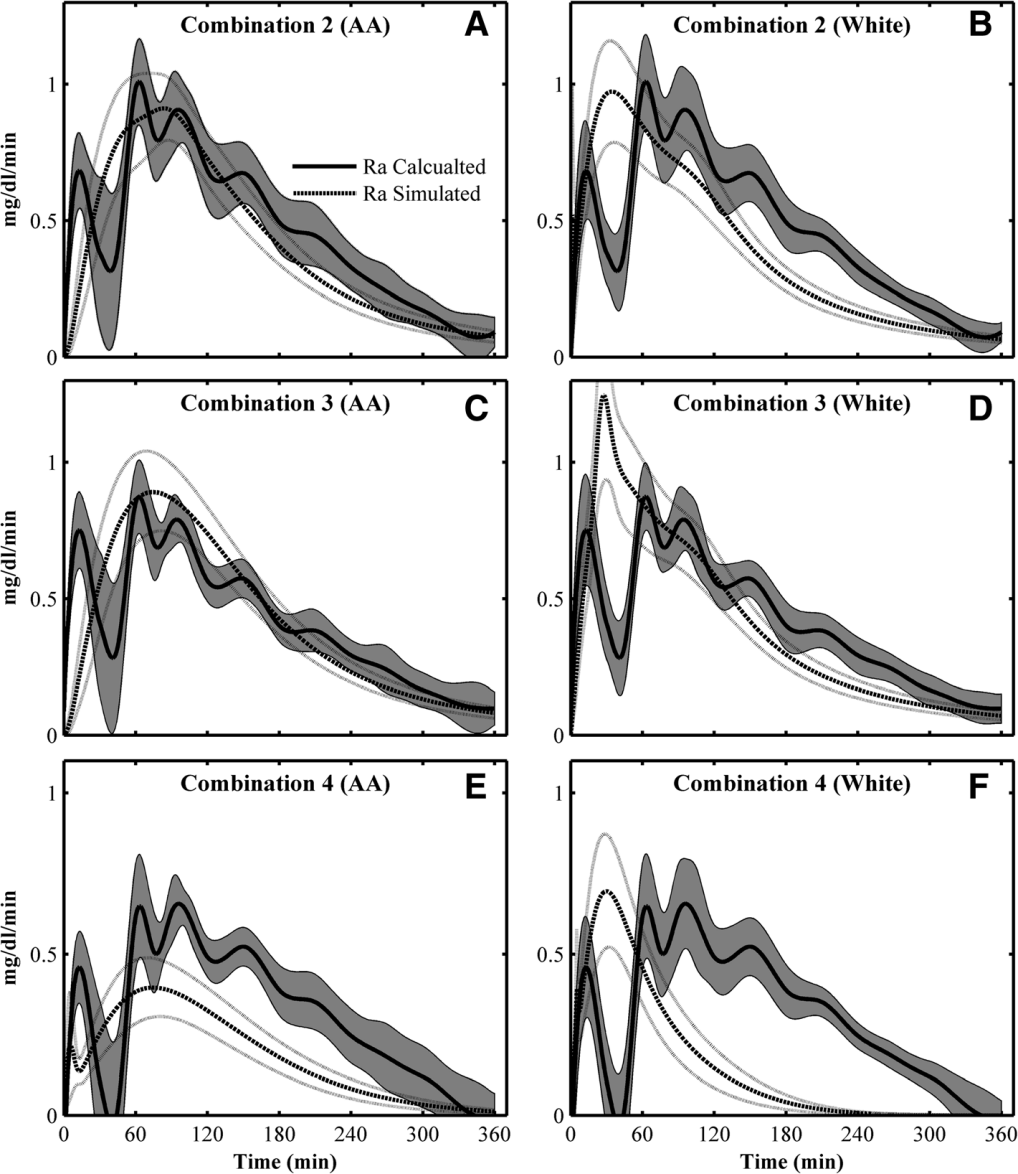
Comparison of the mean calculated glucose Ra and the mean simulated glucose Ra of MOD 1 with Combination 2 (with Ra of glucose and FFA) of **a** AA; **b** white women; Combination 3 (with glucose Ra only, Type I formula) of **c** AA; **d** white women; Combination 4 (with glucose Ra only, type II formula) of **e** AA; **f** white women. The mean and standard error of calculated Ra are represented by a *gray shaded area*; the mean and standard error of simulated Ra are shown with a *dashed line and dotted lines*, respectively

The predicted glucose Ra with MOD 1 according to Combinations 2–4 are compared with calculated glucose Ra in Fig. 5. The predicted glucose Ra with the Type I function matches calculated glucose Ra (Fig. 5 a–d) better than simulated glucose Ra with the Type II function (Fig. 5 e–f). This result is consistent with the better fitting with experimental data, which is not only due to more parameters (3 vs. 2), but also different function characteristics (Type II function leads to underestimation of glucose Ra). The abnormal steep drop in the calculated Ra at ~30-60 min can be attributed to the sparse measurements of glucose in MT, which has dramatic changes during this period and leads to the discontinuous change of glucose change rate (derivative).

### Comparison between groups

Experimental data of AA and white women in IM-FSIGT and MT are shown in Additional file 1: Figure S1. There are no significant differences in glucose, FFA and insulin kinetics in IM-FSIGT. The parameter values of each group obtained under each Combination with three models are shown in Tables 2, 3 and 4 respectively. In specific cases, several parameters are significantly different between AA and white women, e.g., for MOD 1, white women have larger *S*_*I*_ in both Combinations 2 and 3, suggesting more insulin sensitivity (Table 2). The much higher AIRg and DI of AA compared with white women in IM-FSIGT reflects impaired insulin action.

**Table 2:** Comparisons of parameters (mean ± SD) estimated in MOD 1 for each ethnic group in each Combination. Combination 1: without Ra; Combination 2: with Ra for both of glucose and FFA (Type I formula); Combination 3 with Ra of glucose only (Type I formula); Combination 4 with Ra of glucose only (Type II formula). AA: African –American women; white - white women

	Combination 1		Combination 2		Combination 3		Combination 4	
	AA	White	AA	White	AA	White	AA	White
*S*_*G*_	0.014 ± 0.007	0.012 ± 0.008	0.0046 ± 0.0053	0.0064 ± 0.0060	5.6e-3 ± 5.2e-3	6.3e-3 ± 6.4e-3	0.012 ± 0.009	0.0087 ± 0.0079
*G*_*b*_	128.8 ± 41.1	138.1 ± 71.2	186.5 ± 119.9	162.29 ± 108.2	136.4 ± 107.9	156.9 ± 97.8	130.8 ± 70.1	142.8 ± 106.7
*S*_*I*_	4.1e-4 ± 2.1e-4	6.3e-4 ± 4.5e-4	3.1e-4 ± 1.5e-4	5.1e-4 ± 3.0e-4^a^	3.2e-4 ± 1.4e-4	4.9e-4 ± 2.6e-4^a^	0.0077 ± 0.026	5.5e-4 ± 4.3e-4
*C*_*x*_	0.042 ± 0.031	0.053 ± 0.049	0.076 ± 0.045	0.069 ± 0.032	0.12 ± 0.24	0.07 ± 0.03	0.046 ± 0.033	0.091 ± 0.11
*I*_*bx*_	5.1 ± 4.1	5.4 ± 2.8	3.02 ± 3.72	4.54 ± 2.90	4.6 ± 3.5	4.1 ± 2.9	5.2 ± 4.2	5.5 ± 3.2
*l*_*0*_	0.008 ± 0.01	0.017 ± 0.038	0.0026 ± 0.0032	0.003 ± 0.0028	0.006 ± 0.007	0.0017 ± 0.0031^a^	0.0086 ± 0.0092	7.4e-4 ± 1.3e-4^a^
*l*_*2*_	0.14 ± 0.2	0.12 ± 0.14	0.12 ± 0.25	0.048 ± 0.077	0.12 ± 0.21	0.052 ± 0.085	0.089 ± 0.15	0.049 ± 0.067
*X*_*2*_	14.1 ± 15.3	14.1 ± 15.4	12.7 ± 14.18-	14.1 ± 15.6	12.7 ± 15.6	13.6 ± 15.3	13.1 ± 15.7	12.6 ± 14.1
*C*_*f*_	0.092 ± 0.085	0.13 ± 0.19	0.035 ± 0.027	0.029 ± 0.012	0.047 ± 0.25	0.031 ± 0.010^a^	0.063 ± 0.044	0.029 ± 0.012^a^
*K*_*Cl*_	25.3 ± 19.5	15.3 ± 20.3	23.32 ± 18.61	12.1 ± 17.1	21.7 ± 21.5	7.9 ± 11.9^a^	18.6 ± 16.7	6.1 ± 13.6^a^
*m*_*G*_	-	-	118.4 ± 32.96	101.9 ± 37.1	124.9 ± 29.7	107.1 ± 40.7	-	-
*σG*	-	-	0.67 ± 0.29	1.1 ± 0.52^a^	0.66 ± 0.32	0.90 ± 0.42	-	-
*Δ*_*G*_	-	-	65.9 ± 33.6	63.2 ± 24.3	67.9 ± 30.6	65.5 ± 26.6	-	
*σ*_*F*_	-	-	0.61 ± 0.38	0.45 ± 0.31	-	-	-	-
*Δ*_*F*_	-	-	0.32 ± 0.21	0.36 ± 0.19	-	-	-	-
*ϕ*_*G*_	-	-	-	-		-	68.3 ± 41.7	58.1 ± 40.1
*τ*_*G*_	-	-	-	-	-	-	68.4 ± 49.1	33.1 ± 27.8^a^

^**a**^: indicate significantly different between AA and white women in each Combination

**Table 3:** Comparisons of parameters (mean ± SD) estimated in MOD 2 for each ethnic group in each Combination. Combination 1: without Ra of glucose and FFA; Combination 2: with both of Ra of glucose and FFA (Type I formula); Combination 3 with Ra of glucose only (Type I formula); Combination 4 with Ra of glucose only (Type II formula). AA: African –American women; white - white women

	Combination 1		Combination 2		Combination 3		Combination 4	
	AA	White	AA	White	AA	White	AA	White
*S*_*G*_	0.015 ± 0.006	0.014 ± 0.007	0.0061 ± 0.0057	0.0081 ± 0.0059	6.5e-3 ± 5.6e-3	7.2e-3 ± 46.3e-3	0.011 ± 0.0058	8.9e-3 ± 7.4e-3
*G*_*b*_	129.5 ± 25.6	165.9 ± 63.1	189.7 ± 92.3	170.54 ± 79.5	179.3 ± 84.8	170.5 ± 74.5	119.9 ± 45.9	194.2 ± 103.9^a^
*S*_*I*_	4.1e-4 ± 2.3e-4	5.9e-4 ± 2.8e-4	3.6e-4 ± 1.7e-4	5.9e-4 ± 4.7e-4	3.9e-4 ± 2.0e-4	5.5e-4 ± 2.6e-4	4e-4 ± 2e-4	5.8e-4 ± 3.9e-4
*C*_*x*_	0.41 ± 0.029	0.042 ± 0.023	0.072 ± 0.071	0.057 ± 0.029	0.11 ± 0.19	0.053 ± 0.019	0.042 ± 0.024	0.059 ± 0.038
*I*_*bx*_	5.11 ± 3.82	3.24 ± 3.23	5.63 ± 5.33	3.47 ± 3.99	7.4 ± 5.6	5.2 ± 3.8	6.0 ± 6.1	4.5 ± 4.2
*l*_*0*_	0.0095 ± 0.015	0.0036 ± 0.0048	0.033 ± 0.068	0.0092 ± 0.024	0.033 ± 0.061	0.027 ± 0.082	0.0038 ± 0.0044	0.023 ± 0.079
*l*_*2*_	0.98 ± 3.0	0.15 ± 0.21	0.24 ± 0.28	0.17 ± 0.27	0.23 ± 0.32	0.19 ± 0.33	0.13 ± 0.2	0.14 ± 0.25
*X*_*2*_	11.7 ± 12.3	12.8 ± 15.8	12.12 ± 14.81	12.9 ± 14.8	13.4 ± 15.4	13.2 ± 15.9	15.6 ± 16.8	13.1 ± 16.0
*A*_*l*_	2.19 ± 1.01	2.57 ± 0.77	2.13 ± 1.13	2.45 ± 0.82	2.1 ± 0.91	2.4 ± 0.8	2.3 ± 0.99	2.4 ± 0.79
*C*_*f0*_	0.15 ± 0.16	0.19 ± 0.28	0.25 ± 0.34	0.17 ± 0.24	0.23 ± 0.32	0.22 ± 0.35	0.092 ± 0.044	0.13 ± 0.24
*m*_*G*_	-	-	122.31 ± 30.13	100.9 ± 44.9	124.6 ± 31.1	99.4 ± 44.9	-	-
*σG*	-	-	0.66 ± 0.41	0.93 ± 0.65	0.71 ± 0.43	0.78 ± 0.4	-	-
*Δ*_*G*_	-	-	62.7 ± 33.2	59.6 ± 29.7	61.2 ± 32.6	59.3 ± 27.9	-	-
*σ*_*F*_	-	-	0.60 ± 0.38	0.28 ± 0.29^a^	-	-	-	-
*Δ*_*F*_	-	-	0.53 ± 0.39	0.68 ± 0.34	-	-	-	-
*ϕ*_*G*_	-		-	-	-	-	77.2 ± 37.9	83.7 ± 22.1
*τ*_*G*_	-		-	-	-	-	82.9 ± 44.0	64.2 ± 39.4

^a^: indicate significantly different between AA and white women in each Combination

**Table 4:** Comparisons of model parameters of each ethnic group in each Combination with MOD 3. Combination 1: without Ra of FFA. Combination 2: with Ra term of FFA (Type I formula, because MOD 3 only includes FFA kinetics, so only Combinations 1 and 2 are involved). AA: African –American women; White: White women

	Combination 1		Combination 2	
	AA	White	AA	White
*V*_*m*_^*Lip*^	0.54 ± 1.4	3.2 ± 5.8	2.02 ± 4.75	2.35 ± 3.73
*t*_*DelatLip*_	26.9 ± 17.3	36.4 ± 27.0	24.9 ± 14.0	35.7 ± 22.5
*K*_*Lip*_	25.2 ± 24.5	10.2 ± 22.1	10.4 ± 11.9	10.5 ± 13.7
*h*_*LIp*_	4.1 ± 3.8	2.3 ± 2.5	2 ± 2.5	1.8 ± 2.3
*k*_*Rem*_	0.33 ± 1.1	0.67 ± 1.4	0.16 ± 0.45	0.43 ± 1.1
*V*_*m*_^Re*m*^	0.11 ± 0.06c	0.44 ± 0.7	0.25 ± 0.51	0.48 ± 0.66
*t*_*DelatRem*_	12.0 ± 5.9	15.6 ± 6.7	12.6 ± 5.2	14.2 ± 6.6
*K*_*Rem*_	47.4 ± 38.8	42.6 ± 34.1	38.4 ± 37.1	45.4 ± 34.3
*h*_*Rem*_	5.3 ± 3.4	7.8 ± 2.8	6.38 ± 3.56	6.72 ± 3.23
*σ*_*F*_	-	-	0.47 ± 0.35	0.55 ± 0.33
*Δ*_*F*_	-	-	0.24 ± 0.21	0.3 ± 0.19

In MT, the elevations of glucose and insulin are smaller than the values in IM-FSIGT (Additional file 1: Figure S1). White women have a higher peak value (at ~45 min) than AA women, accompanied by significant higher AUC than AA women within 30–60 min (*p* ≤ 0.03), also reflected in the larger coefficient of glucose Ra (*σG*)*.* Predicted Ra of white women also tends to peak earlier than AA women (Fig. 5). These differences suggest that white women may have faster and stronger gastric absorption or specific processes in the liver, or both, which may lead to qualitatively different glucose kinetics - monophasic for white women vs. multiphasic for AA women.

Although the insulin response was relatively smaller in MT compared to IM-FSIGT, the nadir values of FFA in MT were close to the nadir in IM-FSIGT. The relatively smaller insulin regulatory thresholds (*X*_*2*_ in MOD 1 and MOD 2, *K*_*Lip*_ in MOD 3, Tables 2, 3 and 4) than both insulin concentration and simulated insulin action (*X*) (Additional file 1: Figure S6) suggest that lipolysis in adipose tissue is very sensitive to insulin. In IM-FSIGT, higher insulin levels in AA women accompanied lower FFA levels during 0–60 min (Additional file 1: Figure S1, C, E) compared with the responses of white women. However, in MT, although AA women still had higher insulin, FFA levels are significantly higher than white women during 60–180 min (*p* ≤ 0.04). This reversal could, perhaps, be attributed to a different modulation of lipolysis inhibition. In fact, white women tend to reach the nadir faster than AA women (~80 min vs. ~ 130 min). This suggests that the inhibition of lipolysis for AA women may have differences in insulin signaling in adipose tissue.

Predictions for insulin action kinetics (*X*) in IM-FSIGT or MT are compared with insulin data in Additional file 1: Figure S6. The kinetics of *X* in MT are closer to the measured insulin (Additional file 1: Figure S6 C, D). In both IM-FSIGT and MT, the responses of *X* appear to have a time delay compared with insulin, which is consistent with the experimental observations [12].

### Model comparison

Those models under four Combinations are compared with the Bayes information criterion (BIC) in Table 5. Clearly, when both glucose and FFA data are involved, MOD 1 produces a better fit than MOD 2, specifically for Combination 3 (Type I function for glucose Ra, no FFA Ra). The root mean square error (RMSE) of each model and each Combination are shown in Table 6, also verifying upon results. The results obtained based on addition combinations (S1–S3) show that there is no significant difference for the parameters between two ethnic groups in those specific conditions, only FSIGT (S1) or only MT (S2, S3).

**Table 5:** Model comparison using Bayes information criterion (BIC)^a^

Model	Combination	AA	White
MOD 1	1	361.9 ± 81.7	338.9 ± 62.9
	2	345.7 ± 93.1	335.7 ± 69.8
	3	320.5 ± 95.5	307.1 ± 67.5
	4	328.4 ± 87.8	316.8 ± 64.1
MOD 2	1	437.4 ± 84.7	382 ± 56.9
	2	420.7 ± 83.3	360.7 ± 78.4
	3	401.1 ± 74.5	337.51 ± 76.6
	4	405.2 ± 84.0	343.6 ± 61.6

^a^: MOD 3 only covers FFA kinetics, so it cannot be compared with MOD 1 and MOD 2 directly

**Table 6:** Model comparison of goodness of fit. The value is the root mean square error (RMSE). In the calculation, the residual is normalized by the variance of raw data

Combination	Description	Model	Glucose		FFA	
			FSIGT	MT	FSIGT	MT
1	No Ra	1	226.1	254.2	306.4	137.9
		2	284.1	214.2	860.2	138.7
		3	-	-	264.7	122.5
2	Both Ra (Type I)	1	179.2	104.1	378.6	146.8
		2	326.3	114.3	471.9	165.1
		3	-	-	289.6	121.9
3	Only Ra glucose	1	182.5	100.1	239.3	135.5
		2	298.8	119.4	527.8	165.4
	(Type I)	3	-	-	-	-
4	Only Ra glucose (Type II)	1	208.6	203.2	219.7	129.5
		2	266.5	149.6	846.1	148.8
		3	-	-	-	-

## Discussion

The primary aims of this study were to define a simple function to estimate the appearance rate (Ra) of glucose and FFA in MT and to refine an existing model of FFA and glucose kinetics and test its suitability for modeling post-prandial FFA and glucose. As an application, we demonstrated how the contributions of Ra of glucose or FFA on the corresponding kinetics could be quantitatively evaluated. Our new model provides a practical approach to evaluate glucose kinetics in MT.

### Effect of Ra on glucose and FFA kinetics

As expected, we found that glucose Ra plays an essential role in glucose kinetics in MT. Without glucose Ra (Combination 1), the predicted glucose responses completely fail to match the data (Fig. 1 e–f). This is not a surprise, of course, but even the form of the glucose Ra matters: the model predictions based on the Type II function were not satisfactory (Fig. 4, e–f). The fit for white women (Figs. 2 and 3, f) was better than AA women (Figs. 2 and 3, e). Our results suggest that for individuals with monophasic glucose responses in MT, a simple function (Type I, similar to a log-normal functional form) can be used to model glucose Ra in MT (Figs. 2, 3f), making it easier to evaluate glucose kinetics with fewer parameters.

In contrast, for all three models, the simulations with or without introduction of FFA Ra had relatively small differences (Fig. 1 g–h, vs. Figs. 2, 3 and 4, g–h), suggesting that the effect of FFA Ra on post-prandial FFA in MT is limited. This in consistent with studies showing that dietary FFA will not appear in plasma until after ~2 h [22]. Because FFA responses in IM-FSIGT and MT are quite similar, lipolysis and clearance between FSIGT and MT should have similar magnitudes, in agreement with model predictions (Additional file 1: Figure S7, A vs. C, B vs. D). Model simulated FFA Ra did not reach comparable levels with lipolysis and clearance until after 300 min (Additional file 1: Figure S7, C–D). The Ra of FFA can therefore be neglected in modeling post-prandial kinetics.

### Comparison of model behaviors

Model performance is dependent on both of goodness-of-fit and model complexity. BIC was used to compare models in various Combinations, in which MOD 1 under Combination 3 (with Type I glucose Ra) is better than others. Although MOD 3 has better fits for FFA, it only cover FFA data and cannot be directly compared with MOD 1 and MOD 2. The comparison of root mean square error (Table 6) under each condition of three models also reflects those results.

In MOD 3, plasma insulin is applied directly with a specific time delay for lipolysis or clearance. This approach has been utilized in other models as well [17]. However, for peripheral tissues, the effects of insulin on glucose or FFA are generally dependent on the activation of insulin signaling, which then impacts other processes, e.g., the translocation of GLUT4 in skeletal muscle. Because of distinct time courses and magnitudes of insulin signaling and insulin concentration, it may not be physiologically accurate to use the insulin concentration directly, even with a time delay, as suggested by experimental study [12].

The kinetics of the disappearance rate of glucose are different from insulin kinetics [41], suggesting that insulin acts through a insulin action (*X*) as used in MOD 1 and MOD 2. According to a previous study [30], the assumption that glucose and FFA are related to different remote insulin actions did not improve the model while adding complexity.

Mathematical studies have attempted to estimate dynamic glucose Ra [23, 27]. Especially, experimental measured glucose Ra showed that glucose Ra is similar to a log-normal function [38]. In the present study, two functions of glucose Ra are compared (Type I and Type II [40]). Both of them can simulate responses similar to the expected Ra profile of glucose. The two functions have 3 or 2 free parameters, respectively. The results of Type I are much better than the simulations of Type II (Combination 3 vs. Combination 4 for both MOD 1 and MOD 2 (Figs. 2, 3 and 4, e–f), as evident in the improved BIC value that prefers this model. Therefore, with only 3 parameters, the Type I equation is a good choice to estimate glucose Ra in the postprandial Combination for a monophasic glucose responses, compared with the complicated mathematical calculations in some studies [23, 27].

### Approaches to model simplification

In the lipid uptake term, the insulin-independent clearance constant and the maximal clearance rate coefficient were combined together as one parameter (*C*_*f*_, Eq. B6 in Additional file 1). Because FFA is a mixture of fatty acids of different chain lengths, the short and middle chain FFA can enter into peripheral tissue by simple diffusion. Insulin only influences long chain FFA [33, 35, 37, 42]. Our definition balances the roles of different FFA and had little effect on model predictions. The Ra of glucose is simpler than previous studies [25–27].

### Model limitations and future improvement

In agreement with the literature [43], glucose of AA women had multiphasic characteristics (Additional file 1: Figure S1, B). Although simulations using the Type I function work well for white women, but they do not fit data for AA women. Therefore Type I glucose Ra may be inappropriate for specific individuals. Either a better functional form of glucose Ra for multiphasic cases needs to be developed, or a distinct model of glucose-insulin dynamics for MT versus FSIGT which is consistent with the same monophasic Ra of glucose needs to be developed. Clearly, a tracer study would be the appropriate way to test which possibility correctly represents the ethnicity-dependent MT and FSIGT kinetics. The reasons for more complex glucose kinetics in AA women in MT need further study. Predictions of MOD 1 still partly deviate from FFA data in the lipolysis inhibition phase, during which FFA drops more rapidly than predicted by the model. This may be due to parasympathetic activity triggered by the meal.

With the data available, we were not able to address the second limitation of the MM approach (mentioned in the Introduction) in this work, but we note that the meal test MM we developed here may be a better starting point towards an autonomic MM. Such an autonomic system would have parameters for insulin secretion as a function of glucose, but almost certainly would implicitly include a quantification of glucagon dynamics and hepatic glucose output. This is an active area of research and no consensus has appeared to our knowledge.

## Conclusion

In conclusion, we developed a single MM for glucose and FFA that is appropriate for both IM-FSIGT and MT. Data from both IM-FSIGT and MT can be fitted well with this model. The model (MOD 1) attempts to balance complexity and goodness of fit while keeping the mechanisms modeled physiologically plausible. Model simulations show that white women may have higher insulin sensitivity. The Ra of FFA in MT may not play an important role in FFA kinetics. The expression for glucose Ra that we developed, which is similar to a log-normal functional form, provides a way to simplify the estimation of glucose dynamics in MT in some cases.

## Additional file

Additional file 1: Supplemental materials. (DOC 386 kb)
