# Supplementary material for: Modeling glucose and free fatty acid kinetics in glucose and meal tolerance test
Source: Theor Biol Med Model. 2016 Mar 2;13:8. doi: 10.1186/s12976-016-0036-3 (PMC4776401; doi:10.1186/s12976-016-0036-3)
Supplement: Additional file 1: — Supplemental materials. (DOC 386 kb) [file 12976_2016_36_MOESM1_ESM.doc]

# Supplemental materials

Modeling Glucose and Free Fatty Acid Kinetics in Glucose and Meal Tolerance Test

## Yanjun Li, Carson Chow, Amber B. Courville, Anne E. Sumner, Vipul Periwal

### Section A: Additional figures and tables

**Table S1** Demographic and metabolic characteristics.

| Variable | African-Americans  (n = 13) | White  (n = 15) | *P* value |
| --- | --- | --- | --- |
| Age (yr) | 39± 12 | 42±10 | 0.41 |
| Weight (kg) | 90.2±21.6 | 85.1±16.4 | 0.46 |
| Height (cm) | 163.6±5.0 | 168.4±7.4 | 0.07 |
| BMI kg/m^2^ | 33.6±7.2 | 29.8±4.3 | 0.12 |
| WC (cm) | 99.4±16.8 | 100.3±14.4 | 0.98 |
| Hip circumference (cm) | 116.0±18.7 | 113.6±10.8 | 0.58 |
| WHR | 0.86±0.07 | 0.88±0.06 | 0.55 |
| Thigh circumference (cm) | 65.1±10.6 | 57.4±6.3 | 0.07 |
| Fat mass (kg) | 37.5±15.3 | 35.2±11.3 | 0.68 |
| FFM (kg) | 50.3±6.7 | 48.1±5.6 | 0.35 |
| Percent fat (%) | 39.8±8.7 | 39.9±7.4 | 0.94 |
| VAT (cm^2^) | 101.7±77.6 | 104.4±59.3 | 0.82 |
| SAT (cm^2^) | 313.4±188.6 | 264.9±131.4 | 0.66 |
| VAT/SAT ratio | 0.34±0.20 | 0.38±0.15 | 0.43 |
| TG (mg/dl) | 71.±35 | 100±55 | 0.14 |
| HDL cholesterol (mg/dl) | 58±11 | 49±11 | **0.05** |
| AIRg (IM-FSIGT) | 775.4±450.2 | 230.5±189.2 | **0.00** |
| Disposition index (DI) † | 0.22±0.12 | 0.10±0.07 | **0.00** |

† DI is calculated the product of AIRg (IM-FSIGT) and S_I_, S_I_ is obtained in MOD 1 condition 2.

**Table S2** Description of variables and parameter involved in three models.

|  | Description | Unit |  |
| --- | --- | --- | --- |
| Variables and Parameters in MOD 1 and 2 | | | |
| Glucose kinetics | | | |
| S_G_ | Glucose effectiveness coefficient | min^-1^ | Esti.. |
| G_b_ | basal glucose coefficient | mg/dl | Esti.. |
| S_I_ | Insulin sensitivity coefficient | min^-1^ | Esti.. |
| Δ_G_ | Magnitude coefficient of glucose appearance (Type I formula) | Mg/dl | Esti.. |
| m_G_ | Time scale factor of glucose appearance (Type I formula) | Min | Esti.. |
| σ_G_ | Width factor of glucose appearance (Type I formula) | Unitless | Esti.. |
| *ϕ_G_* | Magnitude coefficient of glucose appearance (Type II formula) | Mg/dl | Esti. |
| τ_G_ | Timescale coefficient of glucose appearance (Type II formula) | Min | Esti. |
| Insulin kinetics | | | |
| C_x_ | Coefficient | min^-1^ | Esti. |
| I_bx_ | Coefficient | μU/ml | Esti. |
| FFA kinetics | | | |
| l_0_ | Basal lipolysis rate | mM/min | Esti. |
| l_2_ | Maximal insulin dependent lipolysis | mM/min | Esti. |
| X_2_ | Insulin dependent lipolysis rate constant | μU/ml | Esti. |
| C_f_ | FFA clearance coefficient (MOD 1 only) | min^-1^ | Esti. |
| K_rem_ | Insulin dependent clearance constant () | μU/ml | Esti. |
| A_lipo_, | Hill constant modulating FFA lipolysis (MOD 1 only) | Unitless | Fixed |
| A_Cl_ | Hill constant modulating FFA clearance (MOD only ) | Unitless | Fixed |
| Δ_F_ | Magnitude coefficient of FFA appearance | mM | Esti. |
| m_F_ | Time scale factor of FFA appearance | Min | fixed |
| σ_F_ | Width factor of FFA appearance | Unitless | Esti. |
| A_l_ | Hill constant modulating FFA lipolysis (MOD 2 only) | min^-1^ | Esti. |
| Cf_0_ | FFA clearance rate coefficient (MOD 2 only) | min^-1^ | Esti. |
|  |  |  |  |
| Free Parameters in MOD 3 | | | |
| $V_{m}^{Lip}$ | Maximal lipolysis | mM | Esti. |
| t_DelatLip_ | Time delay of insulin dependent lipolysis | Min | Esti. |
| K_Lip_ | Lipolysis rate constant | μU/ml | Esti. |
| h_Lip_ | Hill constant | Unitless | Esti. |
| k_Rem_ | Basal clearance rate coefficient | min^-1^ | Esti. |
| $V_{m}^{\mathrm{Re}m}$ | Maximal clearance rate | min^-1^ | Esti. |
| t_DelatRem_ | Time delay of insulin dependent clearance | Min | Esti. |
| K_Rem_ | Insulin-dependent clearance constant | μU/ml | Esti. |
| h_Rem_ | Hill constant | Unitless | Esti. |
|  |  |  |  |

**Table S3:** Comparisons of parameters (mean±SD) estimated in MOD 1 for each ethnic group in Combination S1-S3. Combination S1: only IM-FSIGT; Combination S2: only MT (Type I formula); Combination S3 only MT (Type II formula);. AA: African –American women; white - white women.

|  | Combination S1 | | Combination S2 | | Combination S3 | |
| --- | --- | --- | --- | --- | --- | --- |
|  | AA | White | AA | White | AA | White |
| *S_G_* | 0.028±0.055 | 0.034± 0.053 | 0.015±00018 | 0.011±0.019 | 0.021±0.025 | 0.029± 0.036 |
| *G_b_* | 167.5±126.1 | 142.2±100.2 | 175.6±122.1 | 151.1±101.6 | 161.3±106.4 | 151.3±98.6 |
| *S_I_* | 3.6e-4±2.5e-4 | 5.1e-4±2.9e-4 | 5.1e-4±6.2e-4 | 7.1e-4±8.6e-4 | 3.3e-4±2.1e-4 | 4.7e-4±3.9e-4 |
| *C_x_* | 0.075±0.043 | 0.06±0.02 | 0.14±0.26 | 0.26±0.4 | 0.092±0.063 | 0.081±0.042 |
| *I_bx_* | 3.2±4.2 | 3.6±3.1 | 4.3±3.8 | 5.8±2.2 | 3.9±2.9 | 4.2±4.1 |
| *l_0_* | 0.009±0.005 | 0.011±0.009 | 0.006±0.008 | 0.007±0.08 | 0.008±0.003 | 0.009±0.011 |
| *l_2_* | 0.16±0.17 | 0.046±0.02 | 0.22±0.35 | 0.14±0.25 | 0.21±0.18 | 0.036±0.02 |
| *X_2_* | 9.6±9.2 | 11.7±12.6 | 14.6±16.4 | 11.8±11.2 | 12.5±10.2 | 13.7±11.2 |
| *C_f_* | 0.068±0.12 | 0.033±0.015 | 0.06±001 | 0.16±0.3 | 0.051±0.09 | 0.041±0.021 |
| *K_Cl_* | 13.7±14.0 | 8.5±13.1 | 31.2±18.5 | 24.5±19.3 | 23.7±21.2 | 12.8±10.4 |
| *m_G_* | - | - | 107.±44.3 | 94.3±47.4 | - | - |
| *σG* | - | - | 0.79±0.49 | 0.98±0.63 | - | - |
| *Δ_G_* | - | - | 50.8±36.5 | 51.7±28.1 | - | - |
| *ϕ_G_* | - | - |  |  | 61.2±45.3 | 55.7±42.3 |
| *τ_G_* | - | - | - | - | 72.1±41.6 | 37.8±21.6 |

**Table S4:** Comparisons of parameters (mean±SD) estimated in MOD 2 for each ethnic group in Combination S1-S3. Combination S1: only IM-FSIGT; Combination S2: only MT (Type I formula); Combination S3 only MT (Type II formula). AA: African –American women; white - white women.

|  | Combination S1 | | Combination S2 | | Combination S3 | |
| --- | --- | --- | --- | --- | --- | --- |
|  | AA | White | AA | White | AA | White |
| *S_G_* | 0.010±0.005 | 0.012±0.009 | 0.025±0.033 | 0.023±0.03 | 0.015±0.012 | 0.009±0.012 |
| *G_b_* | 170.8±98.6 | 163.3±46.5 | 160.6±50.1 | 143.1±78.2 | 182.3±102.63 | 168.9±55.3 |
| *S_I_* | 4.4e-4±1.8e-4 | 3.2e-4±2.6e-4 | 4.3e-4±2.3e-4 | 4.4e-4±3.3e-4 | 4.7e-4±2.4e-4 | 3.6e-4±2.8e-4 |
| *C_x_* | 0.03±0.02 | 0.056±0.087 | 0.028±0.043 | 0.06±0.08 | 0.02±0.02 | 0.046±0.063 |
| *I_bx_* | 3.1±3.2 | 4.1±3.9 | 8.6±6.6 | 10.9±6.1 | 3.6±4.1 | 3.6±3.1 |
| *l_0_* | 0.0045±0.0046 | 0.0033±0.004 | 0.003±0.004 | 0.006±0.003 | 0.006±0.002 | 0.007±0.004 |
| *l_2_* | 0.29±0.32 | 0.56±0.5 | 0.17±0.21 | 0.32±0.51 | 0.29±0.32 | 0.42±0.45 |
| *X_2_* | 14.1±13.6 | 8.2±8.0 | 18.2±27.6 | 9.2±12.7 | 15.7±12.8 | 10.2±9.8 |
| *A_l_* | 2.3±0.8 | 2.2±0.8 | 2.1±0.8 | 1.8±0.9 | 2.6±1.2 | 2.5±1.1 |
| *C_f0_* | 0.15±0.06 | 0.24±0.22 | 0.29±0.23 | 0.26±0.18 | 0.13±0.11 | 0.21±0.26 |
| *m_G_* | - | - | 104.7±42.3 | 96.2±41.4 | - | - |
| *σG* | - | - | 0.72±0.36 | 0.87±0.42 | - | - |
| *Δ_G_* | - | - | 61.9±38.1 | 52.3±32.6 | - | - |
| *ϕ_G_* | - | - | - | - | 71.6±32.3 | 81.9±32.4 |
| *τ_G_* | - | - | - | - | 84.2±42.1 | 66.7±33.8 |

**Figure S1**

**Figure S1** Comparison of the experimental data for the two ethnic groups in IM-FSIGT and MT. Plasma glucose in A) FSIGT and B) MT; plasma insulin in C) FSIGT and D) MT. Plasma FFA in E) FSIGT and F) MT. Plasma triglyceride (TG) in MT is shown as an inset of F. Experimental data are mean±SE. Blue solid line – African American women (AA); green dashed line – white women (White).

**Figure S2**:.

**Figure S2**: Mean fractional residuals from comparing data and models in Combination 1 (without Ra) for A) Glucose in FSIGT of A) AA women; B) white women ; FFA in FSIGT of C)AA women; D) white women; Glucose in MT of E) AA women; F) white women; FFA in MT of G) AA women; H) white women. Solid line –MOD 1; dashed line –MOD 2; dotted line –MOD 3.

**Figure S3**

**Figure S3** Mean fractional residuals from comparing data and models in Combination 2 (with Ra of glucose and FFA) for A) Glucose in FSIGT of A) AA women; B) white women; FFA in FSIGT of C) AA women; D) white women; Glucose in MT of E) AA women; F) white women; FFA in MT of G) AA women; H) white women. Solid line –MOD 1; dashed line –MOD 2; dotted line –MOD 3.

**Figure S4**

**Figure S4:** Mean fractional residuals from comparing data and models in Combination 3 (with glucose Ra only, Type I function) for Glucose in FSIGT of A) AA women; B) white women; FFA in FSIGT of C) AA women; D) white women; Glucose in MT of E) AA women; F) white women; FFA in MT of G) AA women; H) white women. Solid line –MOD 1; dashed line –MOD 2.

**Figure S5**

**Figure S5:** Mean fractional residuals from comparing data and models in Combination 4 (with Ra of glucose only, Type II function) for Glucose in FSIGT of A) AA women; B) white women; FFA in FSIGT of C) AA women; D) white women; Glucose in MT of E) AA women; F) white women; FFA in MT of G) AA women; H) white women. Solid line –MOD 1; dashed line –MOD 2.

**Figure S6**

**Figure S6**: Average of experimental data of insulin and model simulations of remote insulin in FSIGT for A) AA women B) white women; in MT for C) AA and D) white women. Simulations are generated by MOD 1 in Condition 2. Experimental data: mean±SE. Red solid line, average simulated remote insulin.

**Figure S7**

**Figure S7**: Model predicted each flux associated with FFA kinetics simulated by MOD 1 in FSIGT for A) AA; B) white women; in MT for C) AA; D) white women. Red Solid line – Ra of FFA; blue dashed line – lipolysis; dotted line –FFA clearance.

**Figure S8:** Mean individual simulations obtained in Combination S1 (only IM-FSIGT) and comparison with the mean experimental data for Glucose A) AA; B) white women; FFA of C) AA; D) white women; Open square - experimental data (mean±SE); solid line - mean simulation of MOD 1; dashed line - mean simulation of MOD 2.

**Figure S9:** Mean individual simulations obtained in Combination S2 (only MT, Type I function) and comparison with the mean experimental data for Glucose A) AA; B) white women; FFA of C) AA; D) white women; Open square - experimental data (mean±SE); solid line - mean simulation of MOD 1; dashed line - mean simulation of MOD 2.

**Figure S10:** Mean individual simulations obtained in Combination S3 (only MT, Type II function) and comparison with the mean experimental data for Glucose A) AA; B) white women; FFA of C) AA; D) white women; Open square - experimental data (mean±SE); solid line - mean simulation of MOD 1; dashed line - mean simulation of MOD 2.

### Section B Description of Minimal Models

This mathematical MM was developed to simulate the kinetics of plasma glucose, FFA and insulin during IM-FSIGT and MT. We assume that insulin can modulate plasma glucose or FFA metabolism via remote compartments (i.e., insulin action). Glucose and FFA share the same remote insulin action . This simplifies model structure and reduces the number of parameters . In MT simulations, the Ra of glucose or FFA were incorporated into corresponding dynamic equations, representing the rate of glucose or FFA entering the circulation after digestion and absorption of a diet.

In IM-FSIGT, the dynamic equations of glucose and remote insulin were

$\frac{dG_{FSIGT}}{dt}=S_{G}G_{b}-(S_{G}+S_{I}X_{FSIGT})G_{FSIGT}$ (B1)

$\frac{dX_{FSIGT}}{dt}=C_{x}[I_{FSIGT}(t)-X_{FSIGT}-I_{bx}]$ (B2)

where *G* is the plasma glucose concentration; *X* represents insulin action in the remote compartment; *I(t)* is the plasma insulin concentration measured at specific time points. During model simulation, the dynamics of *I(t)* at intermediate times were obtained by interpolation of the measured values. *S_G_*, *G_b_*, *S_I_*, *C_x_*, and *I_bx_* are free parameters.

The dynamic equation of FFA is modified in the current model. Plasma FFA kinetics is determined by production and removal (clearance) rates. The production rate corresponds to the lipolysis rate in adipose tissue, which is inhibited by remote insulin. The removal of FFA from plasma to peripheral tissues (FFA clearance) is assumed to be upregulated by insulin as well . Both the lipolysis (*Lipo*) and clearance (*Cl*) rates of FFA were expressed as Hill functions.

$\begin{matrix} \frac{dF_{FSIGT}}{dt}=Lipo_{FSIGT}-Cl_{FSIGT} \\ Lipo_{FSIGT}=\left[l_{0}+\frac{l_{2}}{1+(X_{FSIGT}/X_{2})^{A_{lipo}}}\right]\text{ Cl}_{FSIGT}=C_{f}\left[1+\frac{\left(X_{FSIGT}/K_{Cl}\right)^{A_{Cl}}}{1+\left(X_{FSGT}/K_{Cl}\right)^{A_{Cl}}}\right]F_{FSIGT} \end{matrix}$(B3)

where *F* is the plasma FFA concentration. *l_0_*, *l_2_*, *X_2_*, *C_f_*, and *K_Cl_* are parameters.

Compared with FSIGT, the primarily physiological difference in MT is that both glucose and FFA in blood have two sources. The first source is the same as FSIGT, either from hepatic tissue (for glucose) or from adipose tissue (FFA), which is insulin-inhibited. The second source is the meal. Glucose and FFA components from the meal appear in the blood after digestion and absorption. Therefore, in the MM, an additional appearance rate (*Ra*) can be introduced. Based on the above equations in IM-FSIGT, the model equations in MT can be expressed as

$\frac{dG_{MT}}{dt}=S_{G}G_{b}-(S_{G}+S_{I}X_{MT})G_{MT}+Ra_{G}(t)$ (B4)

$\frac{dX_{MT}}{dt}=C_{x}[I_{MT}(t)-X_{MT}-I_{bx}]$ (B5)

$\begin{matrix} \frac{dF_{MT}}{dt}=Lipo_{MT}(t)+Ra_{F}(t)-Cl_{MT}(t) \\ \text{ }Lipo_{MM}(t)=\left[l_{0}+\frac{l_{2}}{1+(X_{MT}/X_{2})^{A_{lipo}}}\right]\text{ Cl}_{MMT}(t)=C_{f}\left[1+\frac{\left(X_{MT}/K_{Cl}\right)^{A_{Cl}}}{1+\left(X_{MT}/K_{Cl}\right)^{A_{Cl}}}\right]F_{MT} \end{matrix}$(B6)

where *G_MT_*, *I_MT_*, and *F_MT_* are the plasma glucose, insulin and FFA concentration during MT; *X_MT_* is the corresponding remote insulin concentration. *Ra_G_(t)* and *Ra_F_(t)* are the appearance rates of glucose and FFA, respectively, which quantitatively define the fluxes of glucose or FFA generated from diet.

**Section C Comparison of various models and conditions**

To evaluate the effect of appearance rate Ra of glucose or FFA on simulated glucose and FFA kinetics, the above three models are compared under four simulation combinations. Both IM-FSIGT and MT data are used together to determine the unknown parameters.

*Combination 1.* Both of the Ra of glucose and FFA are not introduced in MT.

*Combination 2.* Both of Ra of glucose and FFA are incorporated in the dynamic equation in MT, respectively, which are expressed as Type I function.

*Combination 3*. only Ra of glucose is introduced, expressed as Type I function.

*Combination 4*. only Ra of glucose is introduced as a Type II function.

Based on those results, to completely investigate the parameter stability and simulation responses, addition combinations are involved for MOD1 and MOD 2.

*Combination S1.* Only IM-FSIGT.

*Combination S2.* Only MT, Ra of glucose is expressed as Type I function;

*Combination S3.* Only MT, Ra of glucose is expressed as Type II function;

The behaviors of three models in each condition are compared by Bayes information criterion (BIC) calculated as

$$BIC=SSE+p\ln(n)$$

where *n* is the number of data points, *p* is the number of parameters in each case, SSE is the total sum of squared errors normalized by the variance of data for glucose and FFA, respectively.

References

1. Periwal V, Chow CC, Bergman RN, Ricks M, Vega GL, Sumner AE: **Evaluation of quantitative models of the effect of insulin on lipolysis and glucose disposal.** *Am J Physiol Regul Integr Comp Physiol* 2008, **295:**R1089-1096.

2. Bonen A, Chabowski A, Luiken JJ, Glatz JF: **Is membrane transport of FFA mediated by lipid, protein, or both? Mechanisms and regulation of protein-mediated cellular fatty acid uptake: molecular, biochemical, and physiological evidence.** *Physiology (Bethesda)* 2007, **22:**15-29.

3. Glatz JF, Luiken JJ, Bonen A: **Membrane fatty acid transporters as regulators of lipid metabolism: implications for metabolic disease.** *Physiol Rev* 2010, **90:**367-417.

4. Kazantzis M, Stahl A: **Fatty acid transport proteins, implications in physiology and disease.** *Biochimica et biophysica acta* 2012, **1821:**852-857.

5. Ramos-Roman MA, Sweetman L, Valdez MJ, Parks EJ: **Postprandial changes in plasma acylcarnitine concentrations as markers of fatty acid flux in overweight and obesity.** *Metabolism* 2012, **61:**202-212.

6. Schwenk RW, Holloway GP, Luiken JJ, Bonen A, Glatz JF: **Fatty acid transport across the cell membrane: regulation by fatty acid transporters.** *Prostaglandins Leukot Essent Fatty Acids* 2010, **82:**149-154.
